# Personal Authentication Using Multifeatures Multispectral Palm Print Traits

**DOI:** 10.1155/2015/861629

**Published:** 2015-06-14

**Authors:** Gayathri Rajagopal, Senthil Kumar Manoharan

**Affiliations:** Department of Electronics and Communication Engineering, Sri Venkateswara College of Engineering, Sriperumbudur Taluk, Tamil Nadu 602117, India

## Abstract

Biometrics authentication is an effective method for automatically recognizing a person's identity with high confidence. Multispectral palm print biometric system is relatively new biometric technology and is in the progression of being endlessly refined and developed. Multispectral palm print biometric system is a promising biometric technology for use in various applications including banking solutions, access control, hospital, construction, and forensic applications. This paper proposes a multispectral palm print recognition method with extraction of multiple features using kernel principal component analysis and modified finite radon transform. Finally, the images are classified using Local Mean *K*-Nearest Centroid Neighbor algorithm. The proposed method efficiently accommodates the rotational, potential deformations and translational changes by encoding the orientation conserving features. The proposed system analyses the hand vascular authentication using two databases acquired with touch-based and contactless imaging setup collected from multispectral Poly U palm print database and CASIA database. The experimental results clearly demonstrate that the proposed multispectral palm print authentication obtained better result compared to other methods discussed in the literature.

## 1. Introduction

The field of hand vascular pattern investigation has invited a lot of concentration for forensic and civilian usage as suggested by Jain and Feng [1]. Multispectral palm print technology uses subcutaneous vascular network on the hand to identify personals in biometric application. The pattern of multispectral palm print is unique to each personal, even between identical twins. Therefore, the pattern of multispectral palm print is a highly distinctive feature that may be used for authenticating the personal as suggested by MacGregor and Welford [2]. Multispectral palm print biometric technology is relatively new and it is in the progression of being endlessly refined and developed.

Since the introduction of multispectral palm print technology, a number of efforts have been put to extend to other multispectral pattern technologies such as multispectral finger print retinal pattern. Although multispectral palm print technology is an ongoing immobile research area of biometric system, a large number of units have been installed in many applications such as security, time and attendance, access control, and hospitals, compared to other biometric traits. Vascular pattern technologies afford advantages such as better usability and higher recognition accuracy.

The rest of this paper is structured as follows.  Section 2 presents the details on the survey of the proposed work.  Section 3 presents the details on preprocessing steps for multispectral palm print biometric system and the block diagram of the proposed multispectral palm print system for human identification.  Section 4 describes the proposed multispectral palm print feature extraction methods and classification approach for automated multispectral palm print identification. Experimental results from the proposed and prior approaches are discussed in Section 5. The key solutions are summarized in Section 6.

## 2. Related Work

There are many different features in multispectral palm print image such as the delta point and the principle line and the geometry furthermore multispectral component show significant textural features similar to the features of fingerprints as discussed by Lin et al. [3]. In [4–9] the researchers suggested many multispectral palm print methodologies. Kumar and Prathyusha [10] suggested a new approach using triangulation of multispectral palm print images and extraction of knuckle shape. In [9–11] the researcher suggested various multimodal biometric authentications using multispectral palm print methodology and combined palm print with other traits. In another approach, Wang et al. [12] proposed a hand dorsal vascular approach using partition local binary pattern and validated output using similarity measures. Khan et al. [13] proposed a vascular pattern recognition principal component analysis, which is a lower dimensional image vascular pattern features.

Multispectral palm print recognition methods can be classified into two types' holistic approaches and geometric approaches. Holistic approaches use subspace learning. Various subspaces have been investigated for the multispectral palm print recognition. In [14] the researcher suggested SIFT and LPP and (PCA) principal component analysis are suggested by Wang et al. [15]. In this subspace learning approaches subspace coefficients are generated which is used for recognition during matching. The multispectral palm print images represent vascular structure and therefore the extraction of geometric feature has invited a lot of research focus. Here the ROI extraction is attained using spatial domain filtering, and a number of filters have been investigated. In [16] the researcher suggested SUSAN edge detector, cut-off Gaussian filter is suggested by Toh et al. [17], Gabor filter is suggested by Zhang et al. [18] and orthogonal Gaussian filter is suggested by Hao et al. [19], and Safa et al. [20] proposed behavioral pattern based identification.

The summary of previous work in the literature suggested that not so many researchers have paid attention to use multiple algorithms to increase the recognition accuracy. And the literature proposes the deficient of systematic comparison of different feature representation for the multispectral palm print methodologies; in [16–18] the researcher used constrained images in their research and Hao et al. [19] use contact-free images.

This paper proposes multiple algorithms based multispectral palm print recognition. The proposed approach uses two new methodologies to extract two different multispectral features and obtained improved recognition accuracy. The proposed subspace learning approach using KPCA (kernel principal component analysis) and MFRT (modified finite radon transform) approach offers a computationally efficient and utilizes minimum templates size compared to the existing approaches. The proposed method obtained improved recognition performance as compared to the previous multispectral palm print approaches presented in the literature.

## 3. Proposed Work

A review of previous work on multispectral palm print recognition methodologies presented in the earlier section sketches the importance of comparative performance on the most promising multispectral palm print methodologies. In addition, the earlier efforts have been more concentrated on constraint images rather than contact less images. The key contributions are as follows.

- Here we used a comparative analysis of the proposed methodologies in both constrained and contact less multispectral palm print imaging environment which assures establishing the robustness of proposed method.
- The proposed work thoroughly assesses the recognition performance for multispectral palm print recognition. The recognition performances are very good even under the minimum number of training samples (only one sample for each class). This paper also explored the recognition performance of various approaches using two different databases to access the performance using different approaches.

**Figure 1:**
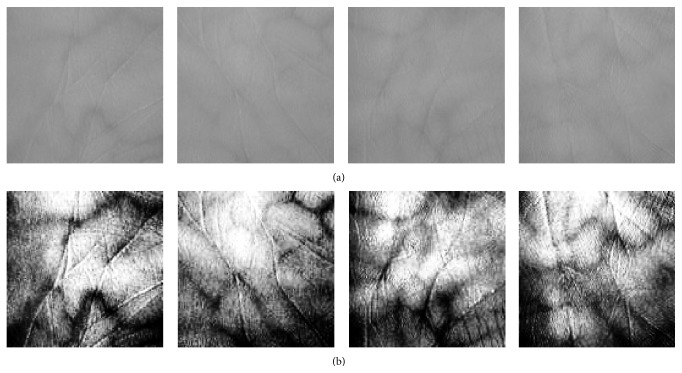
Image enhancement: (a) original ROI image; (b) enhanced image.

The multispectral palm print images in peg-free imaging system presented have many translational and rotational variations. Therefore, accurate preprocessing steps are required to obtain an aligned and stable ROI. The multispectral palm print images are binarized to separate the palm region from the background region. Then distance from the centre location of the binarized palm image to the border of palm image is calculated. Due to the contactless environment, potential scale changes can be quite large, and with the intention of accounting for this variation, it is sensible, to adaptively choose the size and location of the ROI. This can be done using certain image-specific procedures from the palm. To illustrate the multispectral palm print texture pattern more clearly image enhancement is required. From Figure 1 it is clear that the image normalization and enhancement enhanced the fine points and contrast of the ROI images.

## 4. Methodology

**Figure 2:**
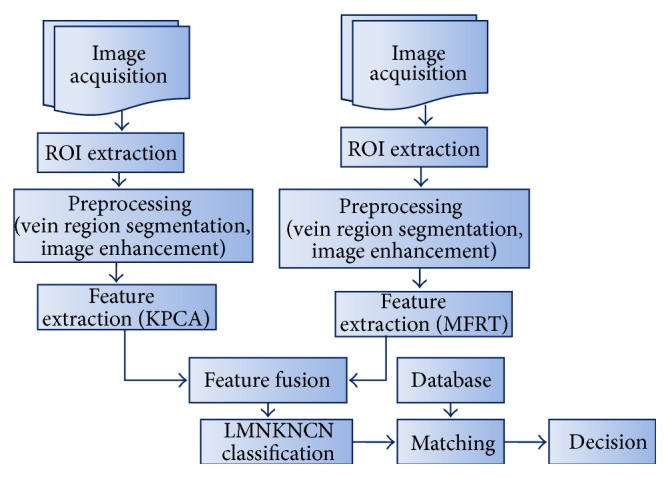
Block diagram for personal identification using multispectral palm print biometric system.

The block diagram of the proposed methodology is shown in Figure 2. The acquired multispectral palm print images are first subjected to preprocessing steps, which extracts the region of interest (ROI). The enhanced and normalised multispectral palm print images are employed with multiple feature extraction algorithms using kernel principal component analysis (KPCA) and modified radon transform (MFRT). The features are fused using feature fusion methodology and features are classified using LMKNCN classification (Figure 5). Matching scores obtained are similar to conventional biometric system.

The preprocessed multispectral palm print image represents curved multispectral patterns, and small line segments, which are rather curved, can approximate these patterns. Therefore, in this research, a new approach is recommended to extract such features. Here, multiple feature extraction using kernel principal component analysis (KPCA) and modified finite radon transform (MFRT) is proposed. The proposed approach can effectively accommodate for more frequent translational, rotational variations, and image distortions.

### 4.1. Kernel Principal Component Analysis (KPCA) Feature Extraction

In principal component analysis, firstly, every eigenvalue and eigenvector are computed and arranged. Then, eigenvectors occurring at the topmost are selected to project the input data. Projecting the input into the selected eigenvectors, the size of the input data significantly decreased. Initially, the mean centre of the image is computed, as *m*′′ is the mean image as given in

(1) $$\begin{array}{c} m^{\prime \prime }=\frac{1}{M^{\prime \prime }}\overset{M^{\prime \prime }}{\underset{i=1}{\sum }}x_{1}, \end{array}$$

where *x*_1_ is a dimensional vector.

Mean centred image (*m*_*j*_) is represented in

(2) $$\begin{array}{c} m_{j}=x_{1}-m^{\prime \prime }. \end{array}$$

Then, covariance matrix is accomplished by

(3) $$\begin{array}{c} C=M^{\prime \prime }M^{\prime \prime T}, \end{array}$$

where *M*′′ represents the composed matrix of the column vectors *m*_*j*_.

Solving *λn*_1_ = *Cn*_1_ eigenvectors and eigenvalues are obtained, assuming *λ* is eigenvector and *n*_1_ is eigenvalue. Consider

(4) $$\begin{array}{c} M^{\prime \prime }M^{\prime \prime T}\left(\;M^{\prime \prime }n\;\right)=\lambda^{\prime }\left(\;M^{\prime \prime }n\;\right). \end{array}$$

It means that the first *M*′′ − 1 eigenvectors (*λ*) and eigenvalues (*n*_1_) could be attained by computing *M*′′*M* ^′′*T*^.

When *M*′′ eigenvalues and eigenvectors are accomplished, the images will be projected onto *L* ≪ *M*′′ dimensions using

(5) $$\begin{array}{c} \Omega^{\prime \prime }={\left[\;n_{1},n_{2},\ldots ,n_{L}\;\right]}^{T}, \end{array}$$

where *Ω*′′ correspond to the projected value. To decide the best picture of a multispectral palm print image, the Euclidean distance can be computed using

(6) $$\begin{array}{c} \epsilon_{k}^{\prime \prime }=\left\|\;\Omega^{\prime \prime }-\Omega_{k}^{\prime \prime }\;\right\|. \end{array}$$

**Figure 3:**
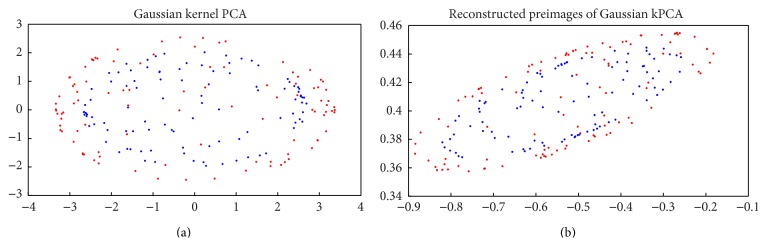
Feature extraction: (a) KPCA feature using Gaussian mapping, (b) restructured image of (a).

At the final step, the minimum assigns the unidentified data into *k* class. KPCA is the same as PCA. In KPCA the input data is nonlinearly mapped and principal component analysis carried out on the obtained data. The mapping is denoted as Φ.  Figure 3 illustrates the feature extraction output of KPCA. After extracting the multiple feature extraction, choosing the suitable classifier plays a main role in getting higher accuracy. For the proposed technique, LMKNCN classification techniques could be used for the enhanced results.

### 4.2. Modified Finite Radon Transform Feature Extraction Method

The radon transform is the integral transform which contains the integral of a function over straight lines. Otherwise, it is the projection of an image along particular direction [20–22]. Let us assume that (*x*_1_, *y*_1_) are the Cartesian coordinates of a point in a 2D image and *I*_1_(*x*_1_, *y*_1_) is the intensity. The 2-dimensional radon transform is represented as *R*_*I*_(*ρ*, *θ*):

(7) RIρ,θ=∫−∞+∞∫−∞+∞I1x1,y1δρ−xcos⁡θ−ysin⁡θdx dy,

where *ρ* is the perpendicular distance of a line from the origin and *θ* is the angle produced by the distance vector. In the projected scheme, we have assumed *θ* to be varying from 0° to 180°. To compute the eigenvalues of the attained radon image, given a matrix *C*, a scalar *λ*_1_ is called an eigenvalue of *C* if there exists a nonzero vector *x*_1_ such that

(8) $$\begin{array}{c} Cx_{1}=\lambda x_{1}. \end{array}$$

Thus, for a square matrix *C* of size *m* × *m*, the eigenvalue equation can be given as

(9) $$\begin{array}{c} Cx_{1}-\lambda Ix_{1}=0, \end{array}$$

where *I* is the identity matrix of size *m* × *m*. To get a nontrivial solution for *x*_1_, determinant value is given as

(10) $$\begin{array}{c} det\left(\;C-\lambda I\;\right)=0. \end{array}$$

**Figure 4:**
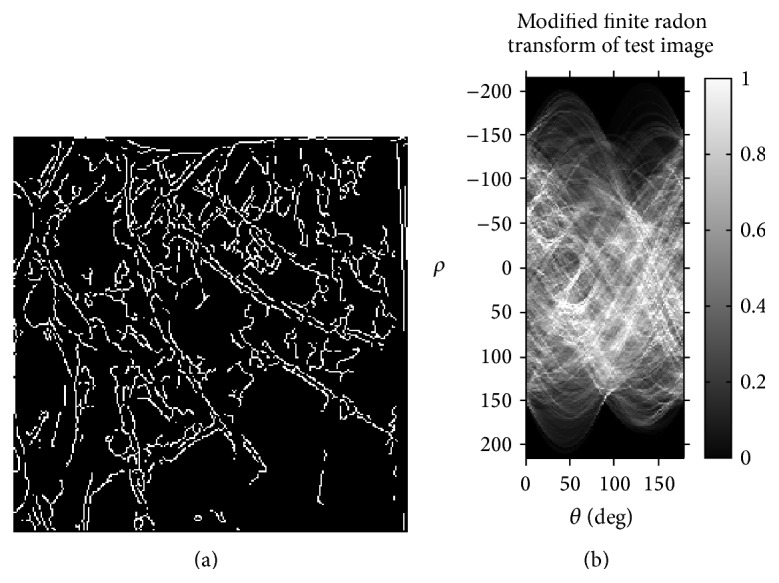
(a) Cropped and canny image and (b) MFRAT feature extraction.

**Figure 5:**
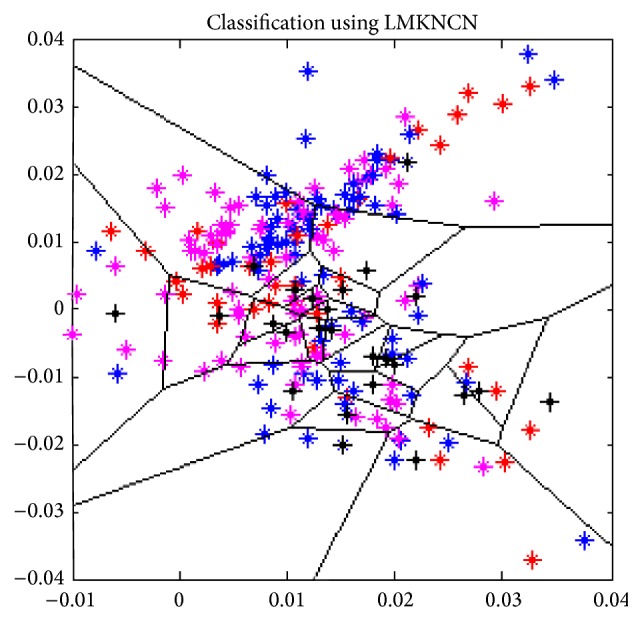
Classification result using LMKNCN.

In the proposed methodology, the angle of projection *θ* is varying from 0° to 180°, and radon transform of an image is represented as a matrix. As the ROI of multispectral palm print image is of same size for all the individuals, the radon transform attained for all the images would be of similar size. However, the attained radon transform may not be a square matrix. In order to make a square matrix we induce equal number of extra rows/columns with all zeroes to radon matrix of each subject. Finally, eigenvalues are calculated for radon matrix.  Figure 4 illustrates the modified finite radon transform output of the cropped canny image.

In the proposed multispectral palm print system, the hand vascular features are the directional information of the hand vascular images. The features are obtained by using the radon projection of a hand vascular image in different angle. Image intensity is calculated for each projection vector. The projection matrix is given as

(11) $$\begin{array}{c} H=\left[\begin{array}{cccc} p_{\theta_{1}}\left(1\right) & p_{\theta_{1}}\left(2\right) & \cdots & p_{\theta_{1}}\left(L\right)\\ p_{\theta_{2}}\left(1\right) & p_{\theta_{2}}\left(2\right) & \cdots & p_{\theta_{2}}\left(L\right)\\ \vdots & \vdots & \cdots & \vdots \\ p_{\theta_{k}}\left(1\right) & p_{\theta_{k}}\left(2\right) & \cdots & p_{\theta_{k}}\left(L\right) \end{array}\right], \end{array}$$

where *k* is the total projection angles and *L* is the number of projection points at *θ*_*i*_(*i* = 1,2,…, *k*). Here the methodologies for multialgorithm multispectral palm print system focus on the feature fusion. Feature fusion is proposed since it uses feature sets that are closest to raw biometric data, which contains rich information compared to matching score and decision level fusion. Therefore, the current investigations on a feature fusion at the feature levels are expected to obtain better recognition accuracy compared to other level fusions.

### 4.3. Local Means *K*-Nearest Centroid Neighbour Classification

Nonparametric classifier KNN is effective, simple, and speedy method to classify data especially in the case of small training sample size. In this technique, Euclidian distance is used as for classifying. To improve the performance of KNN, various methodologies have been proposed out of which newest is LMKNCN. The subsequent section explains the mathematical explanation of LMKNCN.

Let *s* = {*x*_*n*_ ∈ *R* ^*m*^}_*n*=1_^*N*^ be a test set with *M* classes *c*_1_,…, *c*_*M*_ and *s*_*i*_ = {*x*_*ij*_ ∈ *R* ^*m*^}_*j*=1_^*N*^ be a class test set of *c*_*i*_, each consist of training samples *N*_*i*_. In the proposed methodology, when a query pattern is given, its class label can be calculated using the following sequences.

(i) For a given query pattern the set of KNCN using the set *s*_*i*_ of each class *c*_*i*_ can be calculated using
  (12) $$\begin{array}{c} s_{ik}^{NCN}=R_{i}^{NCN}\left(\;x\;\right). \end{array}$$
(ii) Calculate the local centric mean vector *V*_*ik*_^NCN^ from each *c*_*i*_ using *s*_*ik*_^NCN^
  (13) $$\begin{array}{c} V_{ik}^{NCN}=\frac{1}{k}\overset{k}{\underset{j=1}{\sum }}x_{ij}^{NCN}. \end{array}$$
(iii) Compute the distance *d*(*x*, *V*_*ik*_^NCN^) between *x* and *V*_*ik*_^NCN^.
(iv) To obtain the closest distance between local mean vector and query pattern calculate
  (14) $$\begin{array}{c} T=\mathrm{argmin}\;\;d\left(\;x,V_{ik}^{NCN}\;\right). \end{array}$$

## 5. Experimental Classification Results and Analysis

In order to assess the effectiveness of the proposed method for multispectral palm print identification, The researcher evaluated performance using thorough experiments on both contact and contact less based databases. The extraction of multispectral palm print features using Laplacian palm [10], ordinal code [18], and scale invariant feature transform (SIFT) [13] has been investigated in the literature with improved results. Here the investigation compared with all the previous methodologies mentioned in the literature with our proposed methodology. The performance of the proposed multialgorithm multispectral palm print methodology is verified using two databases (i.e.) CASIA [23] (images acquired under 850 nm wavelength) and Poly U [24] (near infrared images) database.

**Figure 6:**
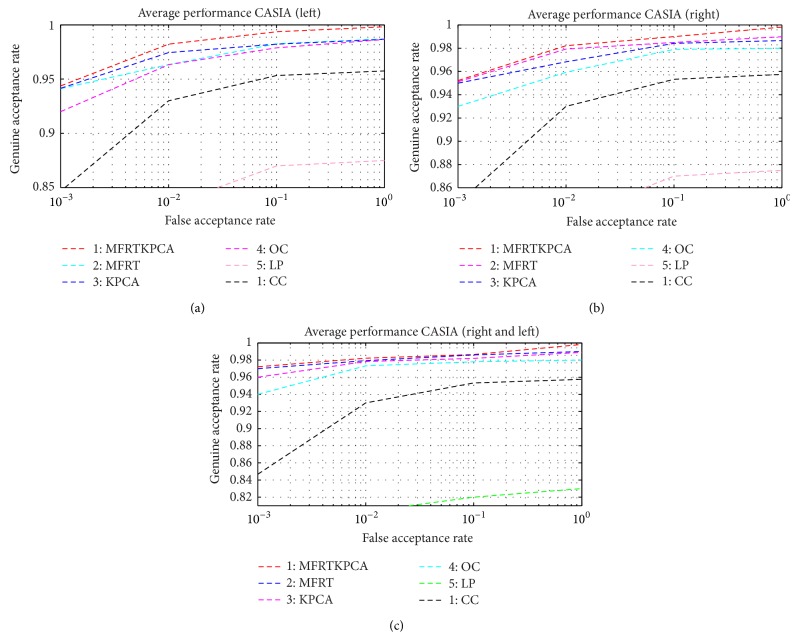
Receiver operating characteristics from the CASIA database: (a) left palm, (b) right palm, and (c) left and right palm.

In this experiment, for the comparative evaluation of the multispectral biometrics authentication using minimum number of training samples per class is considered. This research evaluated the effectiveness of the proposed methodology for various training samples size. This research undergoes three experiments on each of the two databases; firstly the recognition performance from the personals (right and left hands) multispectral palm print images was evaluated. Secondly, to assure the performance researcher combined right hand and left hand multispectral palm print images are considered. The average performance recognition accuracy for various gallery samples from CASIA database is illustrated in Tables 1 [Table tab2]–3. It can be observed from Figure 6 that the proposed multialgorithm (KPCA + MFRT) multispectral palm print approach outperforms other promising methods in all three experiments, especially in the case of a minimum number of samples per class. Unlike the other matching approaches, the proposed method accommodates higher deformation and image variations.

**Figure 7:**
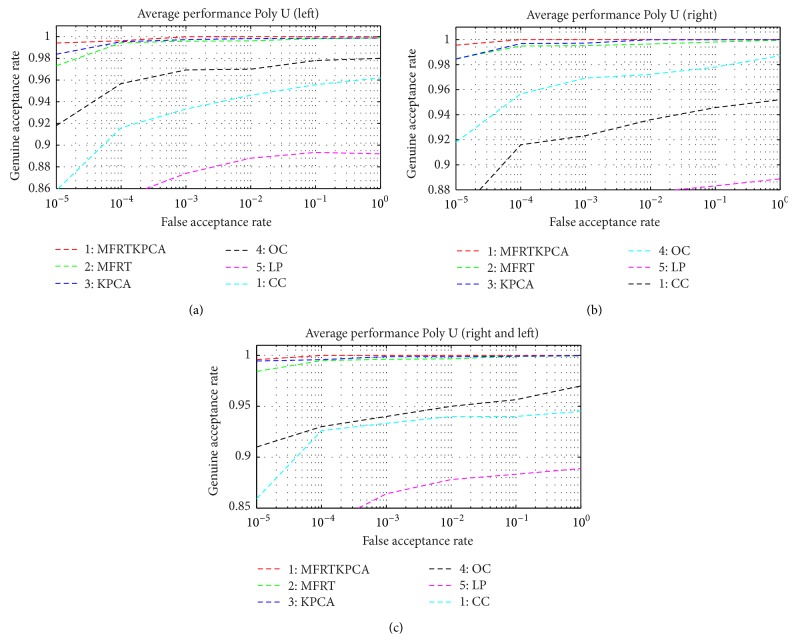
Receiver operating characteristics from the Poly U multispectral palm print database: (a) left palm, (b) right palm, and (c) left and right palm.

Recognition performances from the Poly U multispectral palm print database are illustrated in Tables 4–6. The performance characteristics are obtained from Figure 7. From the above observation, the proposed methodology of multialgorithm multispectral palm print consistently outperforms other promising approaches in all three experiments, even in the case of a smaller number of train images for each class. Unlike other methodologies, the proposed method accommodates higher deformation and image variation and produces best matching. Tables 7 and 8 suggest the performance improvement for the various methodologies with increasing number of samples per class. From the observation, it is suggested that the multialgorithm multispectral approach achieves 0.23% EER for four samples per class using CASIA database. In addition, the same approach achieves 0.006% EER for six samples per class using Poly U multispectral palm print database.

Table 9 summarizes the multispectral palm print approaches reviewed in the literature and suggested the superior recognition performance in the proposed approach. The performance improvement increases with increase in number of training samples on both CASIA and Poly U database. Comparatively improved performance was obtained even when the minimum number of training samples is considered (only one sample for each class).

**Figure 8:**
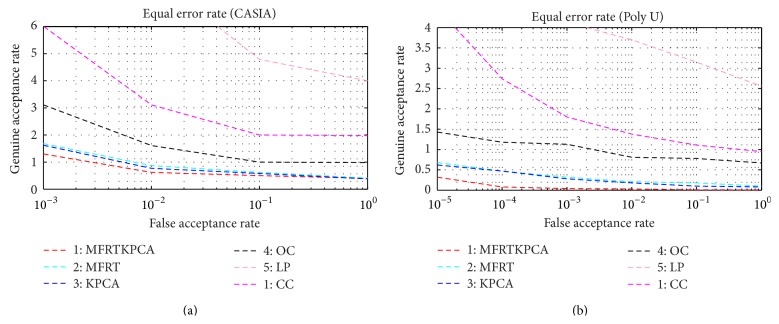
Equal error rate ROC plot: (a) CASIA, (b) Poly U.

The experimental results presented here consistently suggest that the proposed multialgorithm multispectral palm print recognition approach using KPCA and MFRT achieves significantly improved performance over the previously proposed approaches on both peg and non-peg multispectral palm print databases. In order to comparatively determine the performance form the two databases corresponding ROC are shown in Figure 8. It can be observed from the figure that the proposed methodology performs consistently better than the other methods.

## 6. Conclusion

In this paper we have investigated a novel approach for human identification using multispectral palm print images to represent a low resolution multispectral palm print image and to match different multispectral palm print image, we proposed the kernel principal component analysis and modified radon transform methods. We proposed method can effectively accommodate the rotational variation, translational variation, and potential image deformation. We presented an automatic multispectral palm print identification, which can more reliably extract the multispectral palm print features and achieve much higher accuracy than previously proposed multispectral palm print identification methodologies. Our proposed multialgorithm based multispectral palm print identification approach works more effectively and leads to a more accurate performance even with the minimum number of enrolled images (one sample for trainee). We presented rigorous experimental results and compared to the existing methods using two different databases CASIA and Poly U palm print. Here we obtained identification rate of 98.66%, 99.88%, 99.99%, and 100% from the left right multispectral palm print images of the CASIA and Poly U databases, respectively, and correspondingly obtained EER of 0.54, 0.23, 0.01, and 0.006. Performance improvement can be further increased by fusing multispectral palm print image with other suitable traits. Further, a more efficient feature extraction algorithm and classification techniques should be developed to deal with shadow and undesired noise and to decrease the matching score for illegal users and raise the matching score for legal users. Moreover, it should also be competent to reduce the effects from the orientation, nonrigid deformations, and translations of the multispectral palm print image.

## Figures and Tables

**Table 1:** Accuracy measures from CASIA left hand multispectral palm print image.

Number of samples per class	MFRT + KPCA	MFRT	KPCA	Ordinal code	Laplacian code	Comp code	SIFT
1	94.31	94.12	94.15	91.00	75.00	84.67	65.33
2	98.22	96.33	97.44	96.33	83.33	93.00	73.00
3	98.36	98.46	98.22	97.67	87.00	95.33	77.67
4	99.82	98.71	98.82	98.78	87.50	95.75	77.88

**Table 2 tab2:** Accuracy measures from CASIA right hand multispectral palm print image.

Number of samples per class	MFRT + KPCA	MFRT	KPCA	Ordinal code	Laplacian code	Comp code	SIFT
1	95.37	96.25	95.45	93.33	75.33	87.83	68.00
2	98.32	97.38	97.52	95.17	83.00	93.67	75.67
3	98.46	98.40	98.62	97.83	87.83	96.17	80.83
4	99.81	98.90	98.82	98.91	88.96	97.83	83.45

**Table 3:** Accuracy measures from CASIA right and left hand multispectral palm print image.

Number of samples per class	MFRT + KPCA	MFRT	KPCA	Ordinal code	Laplacian code	Comp code	SIFT
1	97.37	97.25	96.45	94.33	80.33	84.83	76.06
2	98.45	97.38	97.52	97.17	81.00	93.69	78.60
3	98.66	98.68	98.72	98.83	82.87	95.17	79.63
4	99.88	98.88	98.79	98.63	83.00	95.30	80.00

**Table 4:** Accuracy measures from Poly U left hand multispectral palm print image.

Number of Samples per class	MFRT + KPCA	MFRT	KPCA	Ordinal code	Laplacian code	Comp code	SIFT
1	99.41	97.48	98.38	91.00	77.67	85.80	75.84
2	99.62	99.43	99.53	95.00	85.13	91.42	78.08
3	99.70	99.58	99.72	96.00	87.40	92.93	78.33
4	99.82	99.61	99.82	97.30	88.80	94.35	78.63
5	99.99	99.81	99.96	97.93	89.33	95.80	79.04
6	100	99.89	99.99	98.93	89.21	96.87	79.28

**Table 5 tab5:** Accuracy measures from Poly U right hand multispectral palm print image.

Number of samples per class	MFRT + KPCA	MFRT	KPCA	Ordinal code	Laplacian code	Comp code	SIFT
1	99.56	98.48	99.45	91.33	76.13	85.96	64.04
2	99.66	99.49	99.60	95.50	85.79	91.42	70.38
3	99.72	99.52	99.67	96.82	87.96	92.71	72.63
4	99.89	99.65	99.85	97.26	88.92	93.29	74.13
5	99.99	99.81	99.99	97.86	89.13	94.67	75.04
6	100	99.95	99.99	98.86	88.92	95.00	75.58

**Table 6:** Accuracy measures from Poly U left and right hand multispectral palm print image.

Number of samples per class	MFRT + KPCA	MFRT	KPCA	Ordinal code	Laplacian code	Comp code	SIFT
1	99.58	98.42	99.45	91.33	64.13	85.96	74.04
2	99.70	99.48	99.58	93.50	76.79	92.42	70.38
3	99.87	99.62	99.80	94.82	83.96	93.11	72.63
4	99.97	99.66	99.88	95.86	87.92	93.29	74.13
5	99.99	99.91	99.92	95.86	88.13	94.07	75.04
6	100	99.96	99.97	97.86	88.92	94.50	75.58

**Table 7:** Equal error rate accuracy measures from CASIA palm print database.

Number of samples per class	MFRT + KPCA	MFRT	KPCA	Ordinal code	Laplacian code	Comp code	SIFT
1	1.39	1.48	1.62	3.11	10.89	6.01	11.55
2	0.68	0.87	0.87	1.61	7.93	3.11	6.99
3	0.54	0.62	0.58	1.00	4.79	2.00	5.19
4	0.23	0.41	0.38	1.98	4.00	1.98	4.00

**Table 8:** Equal error rate accuracy measures from Poly U palm print database.

Number of samples per class	MFRT + KPCA	MFRT	KPCA	Ordinal code	Laplacian code	Comp code	SIFT
1	0.32	0.68	0.62	1.43	10.93	4.48	5.58
2	0.08	0.47	0.47	1.18	6.43	2.74	3.31
3	0.04	0.32	0.28	1.13	4.13	1.80	2.24
4	0.03	0.21	0.18	0.81	3.70	1.38	1.94
5	0.01	0.18	0.10	0.78	3.15	1.11	1.68
6	0.006	0.10	0.08	0.67	2.56	0.95	1.58

**Table 9:** Comparison of multispectral palm print approaches.

Methodology	Classification	Pegs	Size	EER
Gabor features hand vein Kumar and Prathyusha [22]	Euclidean distance	No [CASIA]	106	2.740

Multispectral dorsal vein image Wang et al. [15]	Support vector machine	Yes [Poly U]	250	0.012

Gabor features palm print Zhang et al. [18]	Ant colony optimiation	No [CASIA]	100	3.120

LBP palm vein Kang and Wu [25]	Histogram matching	No [CASIA]	100	0.267

Root sift features palm vein Kang et al. [26]	LBP based mismatching removal	No [CASIA]	105	0.996

Log gabor palm vein Raghavendra and Busch [27]	Spare representation	No [CASIA]	105	8.40

Gabor feature palm vein Kisku et al. [24]	Support vector machine	No [CASIA]	100	3.125

Proposed (KPCA + MFRT)	LMKNCN	No [CASIA]	200	0.230

Proposed (KPCA + MFRT)	LMKNCN	Yes [Poly U]	200	0.006
